# Advances in Parkinson’s Disease Drugs

**DOI:** 10.3390/biom11111640

**Published:** 2021-11-05

**Authors:** Antonio Di Stefano, Lisa Marinelli

**Affiliations:** Department of Pharmacy, “G. d’Annunzio” University of Chieti-Pescara, 66100 Chieti, Italy; l.marinelli@unich.it

Parkinson’s disease (PD) is the second most common neurodegenerative age-related disorder worldwide after Alzheimer’s disease [1,2,3], in which environmental and genetic factors play a pivotal role. PD is characterized by a dopamine deficiency due to dopaminergic neuronal death in the substantia nigra *pars compacta*, and Lewy bodies’ formation, containing alpha-synuclein and ubiquitin [4].

Dystonia, bradykinesia, rigidity, resting tremor, and muscle cramps are the most-reported motor symptoms in PD patients. Moreover, non-motor symptoms, such as mood and sleep disorders, apathy, depression, and cognitive disfunctions are also described [5].

To date, although PD does not have a resolutive treatment, many efforts have been made in the field of drug discovery and delivery to investigate potential useful therapies to mitigate symptoms and provide short-term relief [6]. Levodopa (LD) is considered the most effective available drug to treat the motor symptoms of PD. However, additional medications such as monoamine oxidase B inhibitors, amantadine, anticholinergics, β-blockers, or dopamine agonists, are commonly used [7].

In this Special Issue, five contributions, including four reviews and an original research article, were collected, with the aim of highlighting the latest findings in terms of innovative therapeutic strategies or potential approaches to implement scientific knowledge in the field of neurodegenerative disorders. All the collected papers underline the importance of investing in research regarding the development of novel drugs to improve the clinical framework of PD.

The role of dopaminergic and non-dopaminergic agents was extensively reviewed by De Bello et al. [8]. The investigation focuses on recent receptor ligands that are useful in extending LD response and reducing the undesired side effects. Following the same research line, Carrarini and co-workers [9], beyond summarizing the main PD pharmacological treatments that are suitable in each clinical stage of the disease and provide non-pharmacological treatment-supporting methods. They also emphasized the needed to investigate biomarkers and prodromal symptoms for PD progression.

A transversal approach has been examined by Ciulla et al. [10]. They screened the role of food supplements or functional food based on natural compounds, phytochemicals, vitamins, and minerals for their ability to postpone or alleviate the clinical symptoms of PD. These natural and plant-derived compounds laid the foundation for novel therapeutic approaches. Indeed, most of the examined compounds revealed antioxidant and anti-inflammatory properties, which are essential for counteracting the oxidative stress and inducing dopaminergic neurons’ neuroprotection.

Further contributions consider therapeutic strategies to reduce oxidative stress and metal dyshomeostasis, conditions that are commonly correlated with PD.

Tosato et al. [11] report a list of 800 compounds involved in the management of PD. Among the investigated entities, 250 compounds were considered for their possible metal-chelating properties towards ions that were extensively involved in the loss of metal homeostasis balance in PD.

Moreover, Di Stefano et al. describe novel sulfur- and selenyl-L-Dopa derivatives named **SP1–6** [12]. Among the investigated compounds, **SP6** revealed significant antioxidant and protective activities, counteracting the neurotoxic effect of 6-hydroxydopamine and H_2_O_2_ in a RA/PMA-differentiated SY-SH5Y neuroblastoma cell line. **SP6′s** biological activity could be correlated to its ability to restore selenium deficiency, which is responsible for the lower cognitive ability and reduced motor functions in PD patients.
